# Symptom Management Preference and Persona Development for Mobile Health Design Targeting Chinese Older Adult Patients With Breast Cancer: Descriptive Qualitative Study

**DOI:** 10.2196/71448

**Published:** 2025-08-26

**Authors:** Danyu Li, Yanlin Zhu, Changrong Yuan, Qingmei Huang, Yang Yang, Zhaohui Geng, Xiaotong Yuan, Fulei Wu

**Affiliations:** 1 School of Nursing, Fudan University, 305 Fenglin Road, Shanghai, 200032, China; 2 Department of Nursing, Fudan University Shanghai Cancer Center, Shanghai, China; 3 Department of Oncology, Shanghai Medical College, Fudan University, Shanghai, China; 4 School of Nursing, Shanghai University of Traditional Chinese Medicine, Shanghai, China; 5 International Medical Services, Peking Union Medical College Hospital, Beijing, China

**Keywords:** qualitative, mHealth, preference, symptom management, older adults, personas

## Abstract

**Background:**

Mobile health (mHealth) for breast cancer care can greatly benefit patients’ symptom management. Although research supports the effectiveness of mHealth, older adult patients with breast cancer often face difficulties using it, hindering them from accessing effective symptom management possibilities. Understanding the preference for mHealth among this population is crucial for providing insights into effective mHealth design.

**Objective:**

This study aimed to better understand the symptom management preference using mHealth for Chinese older adult patients with breast cancer and use the approach of personas to inform the mHealth design.

**Methods:**

This was a descriptive qualitative study. In total, 17 patients with breast cancer aged 60 years and older were recruited from tertiary hospitals in Shanghai, China, using purposive sampling. Data were collected through one-on-one interviews. Content analysis was used to identify the factors that influence participants’ symptom management preference using mHealth. The categories of influencing factors of preference informed the persona template and guided the development of the persona.

**Results:**

We identified 3 major categories affecting participants’ preference for mHealth, including social interaction patterns, mHealth literacy, and symptoms. The following five personas were developed: (1) Positive Manager, (2) Dependent Parent, (3) Management Isolationist, (4) Image Manager, and (5) Clinician Dependent. We provide insights into how these personas can be used when designing and implementing mHealth for symptom management support.

**Conclusions:**

Key factors influencing symptom management preference using mHealth among Chinese older adult patients with breast cancer and personas developed based on that can foster a better understanding of this population and initiate future mHealth design and implementation.

## Introduction

Breast cancer is among the main illnesses that threaten the lives and health of older adult women in China, with a double incidence peak at 45‐55 and 70‐74 years of age [1]. The mean age for breast cancer diagnosis in China is 45‐55 years, which is considerably younger than that in Western countries, where the average age is between 48 and 50 years. However, due to the current unrestrained population aging, a substantial number of older adult patients with breast cancer may emerge. Although only 16.6% of patients with breast cancer were aged 65 years or older in 2018 in China, the incidence rate is estimated to increase to 27% by 2030 [2], leading to growing concerns regarding the management of older adult patients with breast cancer. According to previous studies, older adult patients with breast cancer experience higher rates of comorbidities, poorer performance status, limited social support, and difficulty with transportation, thus leading to different treatment approaches than those provided for their younger counterparts or undertreatment [3]. Older adult patients with breast cancer may face a complicated recovery process, insufficient information access, and a lack of emotional support [4,5].

Fortunately, mobile health (mHealth), which refers to medical and public health practices supported by mobile devices [6], is a promising tool that supports symptom management by providing unprecedented access to specialist clinical diagnostics and treatment advice [7]. Currently, mHealth is providing substantial improvements in human lifestyle behaviors and chronic condition management, facilitating the popularity and accessibility of health interventions [8,9]. Regarding disease management for patients with breast cancer, mHealth has demonstrated great potential for improving lifestyles, managing symptoms, and providing information and emotional support [10-14].

Integrating mHealth solutions for older adults can be significantly beneficial to this population, but the process faces various challenges [15]. For example, many mHealth tools may not be designed considering age-related dilemmas, such as vision or dexterity limitations, thus causing accessibility and usability problems [16]. Furthermore, the older adults from lower socioeconomic backgrounds may face affordability barriers to smartphone access and internet connectivity [17,18]. Moreover, a lack of tailored mHealth designed specifically for their needs can decrease the older adults’ willingness to use mHealth, preventing them from adapting to technology and receiving the associated benefits [19,20]. Thus, the successful usage and adoption of mHealth are fairly poor in aging patients, accounting for a large proportion of the customer base for innovative health care devices [21].

Considering the importance of understanding the unique needs of older adult patients with breast cancer, we adopt personas to guide analyses of the needs of this population. The concept of persona was first introduced by Alan Cooper in 1999 [22]. It refers to virtual figures that share specific needs and unique personalities and are categorized based on key information about a target group [23]. Personas are detailed personas that embody the characteristics, behaviors, motivations, goals, and pain points of the identified user segments, thereby enabling more precise interventions [24,25]. Personas serve as the main character in a narrative, a scenario-based approach to intervention design that iteratively generates concepts (in the envision phase), provides feedback to enhance design coherence and appropriateness (in the refine phase), and provides a powerful communication tool to help developers understand the design rationale and prioritize features based on user needs. Considering persona was continuously adopted in different health contexts [20], the age-related challenges in the aging population facing mHealth, in part, can be compensated for by designing senior-friendly mHealth devices using persona.

The specific needs related to mHealth for improving symptom management among Chinese older adult patients with breast cancer have received little focus, and the insufficient use of personas requires further exploration. We typically focus on patients undergoing chemotherapy in this study because of the long duration and cumulative nature of this treatment phase, which is characterized by consistent and increasingly severe side effects such as fatigue, nausea, and immunosuppression. Understanding these patients’ unique challenges is essential for developing effective mHealth solutions to improve their symptom management and overall quality of life. Therefore, this study aims to (1) understand the symptom management behavior and preference for mHealth among this population using qualitative data, (2) develop personas representing patients with different symptom management behavior and preference for mHealth, and (3) provide guidance for integrating personas into the design of mHealth.

## Methods

### Sampling

This study was conducted from April 2022 to February 2023 at the Fudan University Shanghai Cancer Center and Huadong Hospital in Shanghai, China. The purposive sampling method was adopted to enroll participants. The inclusion criteria were as follows: (1) diagnosed with primary breast cancer, (2) aged 60 years and older, (3) undergoing chemotherapy after breast cancer surgery or within 2 weeks of chemotherapy ending, (4) expected survival time of more than 6 months, and (5) know about their own disease status and can express their thoughts and options. The exclusion criteria were as follows: (1) patients currently receiving radiation therapy and (2) patients with vital organ dysfunction. The sample size was guided by the principle of data saturation. Interviews were discontinued after a minimum of 10 sessions, once no new themes emerged across 3 successive interviews [26].

### Data Collection

Eligible participants were identified by nurse leaders in the breast oncology departments based on predefined inclusion and exclusion criteria. After briefly introducing the study aims and obtaining verbal agreement from potential participants to be contacted, the nurse leaders referred the interested individuals to the research team.

Before each interview, participants received a detailed explanation of the study from the research team and signed written informed consent forms. General participant characteristics were then recorded, including age, education, chemotherapy stage, primary carer, and whether living with a primary carer.

Subsequently, one of the authors (YZ) and the corresponding author (FW) conducted the face-to-face interviews. YZ is a nursing undergraduate student who took classes in qualitative study, and FL is a lecturer and experienced qualitative researcher with a research focus on breast cancer and imparted qualitative research methodology in the university. Before the beginning of data collection, YZ was trained by FW, covering the principles of qualitative research, techniques of conducting semi-structured interviews (building rapport, active listening, and probing), and mock interview exercises based on the interview guide. Each interview was audio-recorded and lasted 30‐50 minutes. During data collection, the interviewers summarize and confirm their interpretation of what a participant said during data collection as a member checking to secure the trustworthiness.

Data were collected through semi-structured interviews using open-ended questions. The study aimed to gather qualitative data about participants’ behavior and patterns for symptom management and preference for using mHealth; therefore, the questions were developed carefully around this topic. The original questions were refined after trial interviews with 3 potential patients, and the final interview outlines are presented in Textbox 1. The data from trial interviews were not included in the final data analysis.

Textbox 1. **Interview outline around patients’ symptom management behavior and preference for using mHealth to manage symptoms.**

1. Since starting chemotherapy, how do you feel?
2. What physical, mental, and social interaction changes have you noticed?
3. What are the most dominating symptoms you feel?
4. How do you manage your symptoms? What are the barriers and facilitators of your symptom management experience?
5. Did you get any help to manage your symptoms? Regarding help and resources from your spouse, children, relatives, friends, and health care providers?
6. How do you think about your symptom management skills, attitudes, and experience?
7. How do you think about managing your symptoms using mHealth (eg, knowledge distribution apps, virtual health consultation websites, symptom assessment apps)? Are there any concerns that might discourage you from using mHealth?
8. What features would you find most helpful in mHealth for symptom management?

### Ethical Considerations

This study was reviewed and approved by the Institutional Review Board of Fudan University School of Nursing (IRB no. TYSQ2021-03-05). All participants provided informed consent prior to data collection.

### Data Analysis

The audio-recorded interviews were transcribed within 24 hours after interview completion. All transcripts were managed and coded using NVivo 12. Data were analyzed using qualitative content analysis, and transcripts were coded inductively [27]. By using inductive coding, the data drove the analytical process, ensuring a comprehensive exploration of patterns and themes based on the interview transcripts. This approach allows the researcher to derive insights and meaning directly from the data rather than imposing preconceived coding categories. YZ and DL analyzed the data independently, and the main steps are as follows: (1) immersing in the data by line-by-line reading of the transcript; (2) selecting the unit of analysis; (3) making sense of the data as a whole; (4) open coding; and (5) group and categorization. After open coding, the preliminary codebook was developed and was iteratively refined through constant comparison and discussion between 2 researchers. Codes were then clustered into subcategories and higher-level categories. Intercoder reliability was addressed through regular meetings to compare coding results and resolve discrepancies via discussion until consensus was reached. Investigator triangulation was implemented by involving 2 researchers in data coding and theme development. Discrepancies were resolved through discussion with the other 2 researchers (CY and FW, with 25 y and 10 y of qualitative research, respectively) to reach consensus. Investigator triangulation was used to ensure a comprehensive and trustworthy exploration of subcategories and categories grounded in the interview transcripts.

### Persona Segmentation

After the qualitative data analysis, we identified the categories and subcategories of symptom management behavior and preference for using mHealth to manage symptoms, which could be used as the persona segmentation template. Patients with similar features of categories and subcategories were grouped. Based on these groupings, a persona was created to represent the shared patterns within each subgroup [28]. This ensured that each persona reflected not just individual traits but collective tendencies observed across participants. These personas were sent back to participants for validation to ensure an accurate representation of their characteristics and experiences. Final groupings were refined through team discussions and reviewed by qualitative experts to ensure consistency.

### Persona Creation

Subsequently, we designed the persona skeleton, a layout describing the key information that the persona includes [28]. After finalizing the details of each persona, we used visual icons to highlight their key features and enhance vividness. To further humanize the personas, we assigned them names and incorporated representative quotes to reflect each target group’s personality and context [25]. The visual elements were hand-drawn by the research team to maintain authenticity and engagement.

## Results

### Participants

A total of 17 participants were approached and agreed to participate in the interviews. Participant characteristics are outlined in Table 1. The participants had a mean age of 69.6 (range 61‐80) years. All the participants were female and had undergone radical mastectomy. The most common primary caregivers were their adult children.

**Table 1.** Sociodemographic characteristics of the participants.

No.	Age	Education	Chemotherapy stage	Primary carer	Live with primary carer
N1	72	Primary school	2nd-4th cycle	Children	Yes
N2	73	Illiteracy	5th-8th cycle	Children	Yes
N3	66	High school	2nd-4th cycle	Spouse	Yes
N4	68	Primary school	2nd-4th cycle	Children	Yes
N5	79	Illiteracy	5th-8th cycle	Children	Yes
N6	81	Illiteracy	Chemo-ended	Children	Yes
N7	78	Illiteracy	Chemo-ended	Children	Yes
N8	63	Middle school	5th-8th cycle	Spouse	Yes
N9	61	High school	2nd-4th cycle	Children	No
N10	65	High school	2nd-4th cycle	Children	No
N11	80	Primary school	5th-8th cycle	Children	Yes
N12	66	Middle school	5th-8th cycle	Patient self	NA
N13	65	High school	2nd~4th cycle	Patient self	NA
N14	71	Primary school	Chemo-ended	Children	Yes
N15	64	College	2nd-4th cycle	Children	No
N16	69	High school	Chemo-ended	Children	Yes
N17	63	Middle school	5th-8th cycle	Spouse	Yes

### Symptom Management Behaviors and Preference for Using mHealth to Manage Symptom

We identified 3 categories, 7 subcategories, and corresponding descriptions (Table 2). The results describe the symptom management behaviors and preference for using mHealth to manage symptoms among Chinese older adult breast cancer patients.

**Table 2.** Symptom management behavior and preference for using mHealth to manage symptoms.

Categories	Subcategories	Description
Social interaction patterns	Living conditions	Living with children or spouse who provide 24/7 care or living alone without a specific caregiver
	Social dynamics	Regular interacting with families or friends or completely being isolated and staying at home
mHealth literacy	mHealth access	Having or not having mobile devices (phone or computer) and Internet connection
	mHealth usage	Knowing or not knowing how to use mHealth devices or with amount of experience
	mHealth interest	Being willing or unwilling to interact with others via mobile devices
Symptoms	Symptom diversity	Experiencing many or only a few symptoms simultaneously
	Symptom severity	Experiencing tremendous or bearable symptom burden

### Personas

We classified our findings into 5 personas, with differences in social interaction patterns, mHealth literacy, and symptoms. We typologized the personas as follows: Positive Manager, Dependent Parent, Management Isolationist, Image Manager, and Clinician-Dependent (Table 3).

**Table 3.** Persona overview.

Features	Positive Manager	Dependent Parent	Management Isolationist	Image Manager	Clinician-Dependent
Age	65	66	81	60	70
Education	High school	Primary school	Illiteracy	College	Primary school
Living conditions	Lives with spouse and children providing care	Lives with children who provide 24/7 care	Lives with spouse	Lives with spouse	Lives with children
Social dynamics	Regular interaction with family and friends	Regularly interacts with family members	Isolated, stays mostly at home	Seeks interactions with patient support groups	Regular interaction with clinicians
mHealth access	Has mobile devices and Internet access	Limited access, often relies on children for tech support	No mobile devices or Internet	Has access	Has mobile devices but prefers clinician support
mHealth usage	Actively uses mHealth, searches for professional health information	Uses mHealth only with children’s assistance	Refuses to use mHealth, relies solely on children	Uses mHealth to communicate with peer groups but may need help	Relies heavily on clinicians, limited personal mHealth use
mHealth interest	Interested in web-based clinician interactions and reliable information	Prefers family involvement in mHealth, little personal interest	No interest in using mHealth directly	Prefers engaging with online patient communities for social and emotional support	Trusts only clinician-guided mHealth solutions
Symptom diversity	Experiences multiple symptoms (eg, gastrointestinal issues)	Deals with a mix of symptoms, mainly related to chemotherapy	Experiences chronic pain and discomfort	Focuses on managing self-esteem and emotional symptoms	Multiple symptoms, particularly concerned with treatment side effects
Symptom severity	Manages ssymptoms well but occasionally need support	Struggles with symptom severity	Bears with severe symptoms with willpower	Faces moderate symptom severity but is more focused on appearance	Could bear with symptoms with close clinician guidance
Specific need	mHealth to support chemotherapy-related symptom management, online clinician interaction	mHealth for monitoring by children, simple interface for basic needs	mHealth for children to track appointments and care schedules	mHealth for emotional support, patient-patient communication	mHealth with reliable, clinician-vetted information and consultation options
Design point	Design should focus on providing tips for chemotherapy-related symptom management and ensuring access to reliable professional information, with easy access to online clinician consultations.	Create a simple, user-friendly interface that allows children to monitor patient health and symptom management, ensuring notifications for appointments and medical updates.	Design tools that allow children to remotely manage patient schedules, appointments, and care, with minimal interaction from the patient. Focus on simplifying updates and reminders.	Design should emphasize patient-to-patient interaction for emotional support, with features to help rebuild self-esteem and manage appearance-related concerns.	Provide a trusted, clinician-validated platform with regular updates, symptom management tips, and easy access to clinician consultations.

### Persona of Positive Manager

**Quotes:** *I think I can battle with breast cancer, and I will eventually win. Long days are there (Textbox 2)*.

Textbox 2. **Persona of positive manager.**

*Characteristics:*

- Social interaction patterns: *I live with my caring spouse and supportive children, who play a crucial role in my daily life and health journey. I am deeply grateful for their presence and support, and I cherish every moment with them. Family and friends are my pillars of support. Regular gatherings and conversations keep me mentally engaged and emotionally fulfilled*.
- mHealth literacy: *Having access to mobile devices and the internet has been a game-changer. It empowers me to stay informed about my health and treatment options, which gives me a sense of control. I actively use mHealth apps to educate myself on managing chemotherapy-related symptoms. The ability to connect with healthcare professionals online reassures me and provides personalized advice. I completed high school and have always valued learning. I find comfort in online interactions with healthcare providers. Their expertise and reassurance help me navigate the challenges of treatment with greater confidence*.
- Symptom diversity and severity: *Dealing with various symptoms, such as digestive issues and occasional fatigue, can be challenging. However, learning how to manage them effectively has been empowering. While I generally manage my symptoms well, there are moments when I need extra support. Knowing I can reach out to my family or healthcare team for guidance makes a big difference*.

*Specific preference:* Specifically, I require tailored symptom tracking to monitor chemotherapy-related symptoms accurately. Personalized guidance is essential, offering tips on managing issues like gastrointestinal discomfort and fatigue. Regular updates and alerts keep me informed of symptom changes and treatment updates.

### Persona of Dependent Parent

**Quotes:** *I don’t know what to do and have lost hope for the future. Please tell anything to my children (Textbox 3)*.

Textbox 3. **Persona of dependent parent.**

Characteristics:

- Social interaction patterns: I live with my children, who provide around-the-clock care. We have a close-knit family dynamic, and I rely heavily on them for both emotional support and practical assistance. While I interact regularly with my family members, most of my social interactions revolve around family gatherings and caregiving responsibilities.
- mHealth literacy: I have limited experience with technology and rely on my children to navigate mHealth tools. They assist me in using healthcare apps for basic tasks like appointment scheduling and medication reminders. Understanding complex medical information online is challenging for me, so I prefer simplified explanations and direct guidance from my family.
- Symptom diversity and severity: I manage a mix of symptoms related to chemotherapy, including fatigue and occasional digestive issues. These symptoms can be overwhelming at times, impacting my daily activities and emotional well-being. While I strive to cope independently, I often rely on my children’s support to manage symptom flare-ups and seek medical advice when needed.

Specific preference: I require mHealth tools that simplify symptom monitoring and provide clear instructions for managing chemotherapy side effects. Specifically, I need a user-friendly app that allows my children to track my health status, appointments, and medication schedules effortlessly. Timely alerts and reminders are essential to ensure I adhere to treatment plans and receive prompt medical attention for any symptom changes. Access to reliable information and simplified health advice through mHealth would greatly enhance my ability to manage symptoms and maintain my overall well-being.

### Persona of Management Isolationist

**Quotes:** *I am so severely ill. I don’t want to say anything (Textbox 4)*.

Textbox 4. **Persona of management isolationist.**

Characteristics:

- Social interaction patterns: I prefer solitude and spend most of my time at home, away from social interactions. Living alone with minimal external contact, I find comfort in solitude and independence, handling my health challenges mostly on my own.
- mHealth literacy: I don’t use mobile devices or the internet regularly, which limits my access to mHealth tools. I rely solely on face-to-face interactions with healthcare providers or family members for medical information and support.
- Symptom diversity and severity: Managing chronic pain and discomfort is a daily struggle for me. These symptoms significantly impact my mobility and quality of life, requiring ongoing management strategies to cope independently.

Specific preference: I require healthcare solutions that respect my preference for isolation while providing essential medical support. An ideal mHealth tool would allow minimal digital interaction but provide critical health updates and remote consultations with healthcare professionals as needed. This approach would enable me to maintain my independence while ensuring timely medical guidance and support.

### Persona of Image Manager

**Quotes:** *I haven’t retired. I have to go back to work with a normal body shape (Textbox 5)*.

Textbox 5. **Persona of image manager.**

Characteristics:

- Social interaction patterns: I actively seek interaction with online patient support groups and communities. These platforms provide me with valuable emotional support and information-sharing opportunities, helping me navigate the challenges of chemotherapy with peers who understand my experiences.
- mHealth literacy: I use mHealth apps to connect with patient communities and access reliable health information. I am comfortable navigating online resources and value the empowerment that comes from educating myself about my health and treatment options.
- Symptom diversity and severity: While managing chemotherapy symptoms, I focus on maintaining my appearance and emotional well-being. I deal with moderate symptom severity, such as fatigue and appearance-related concerns, which impact my self-esteem.

Specific preference: I need mHealth tools that facilitate patient-to-patient interactions and provide emotional support. Specifically, I seek platforms that foster community engagement and offer resources for managing appearance-related symptoms. Access to reliable information and peer support online is crucial for enhancing my resilience and emotional health during treatment.

### Persona of Clinician-Dependent

**Quotes:**

*I only rely on health care providers, and I want to contact doctors when I need (Textbox 6)*

Textbox 6. **Persona of clinician-dependent.**

Characteristics:

- Social interaction patterns: I maintain regular interactions with healthcare providers, relying heavily on their expertise and guidance in managing my health. I prioritize direct communication with doctors and healthcare professionals for medical advice and treatment decisions.
- mHealth literacy: While I have access to mobile devices, I primarily use them to facilitate communication with healthcare providers. I prefer direct contact with doctors for accurate medical information rather than relying on digital health tools or online resources.
- Symptom diversity and severity: Managing multiple symptoms related to chemotherapy is a significant concern for me. I depend on clinicians to monitor my symptoms closely and provide tailored treatment plans that address my specific health needs.

Specific preference:

My specific need is a reliable mHealth platform that facilitates direct and timely communication with healthcare providers. I prefer tools that offer secure messaging or virtual consultations with doctors, ensuring I can reach out for medical advice promptly when needed. This direct access to clinicians is crucial for me in managing chemotherapy-related symptoms effectively and receiving personalized care.

## Discussion

### Overview

This study depicted the differences in symptom management behaviors and preference for using mHealth to manage symptoms among the older adult breast cancer patients in China and, based on the differences, built 5 typical personas: Positive Manager, Dependent Parent, Management Isolationist, Image Manager, and Clinician-Dependent. This study outlines the complete process of persona development, including qualitative data collection, persona segmentation, and creation, building upon previous reviews of persona technologies [28]. The strict adherence to the consensus process [28] and early user involvement [29] suggests that our findings may serve as a valuable reference for future persona development and user-centered design.

The qualitative interviews we conducted focused on our target and special population—older adult breast cancer patients. The combination of cancer and aging renders this population particularly vulnerable, as cancer impacts their physical health while aging contributes to increased comorbidities and a decline in overall resilience [30]. Despite previous studies that have primarily focused on the treatment and survival of this population, few have closely examined their self-management behaviors or actively engaged them in the information age to ensure they fully benefit from modern technologies [31]. Notably, we found only 1 previous paper that recognized the need for health technology in chronic disease management [21], and no existing literature specifically addresses the preferences of older adult breast cancer patients. Therefore, our study identifies 3 key categories—interaction patterns, mHealth literacy, and symptom diversity and severity—that revealed the self-management behavior patterns and preference of mHealth for symptom management of older adult breast cancer patients. This research addresses a critical gap in the literature, highlighting the importance of understanding these factors to enhance mHealth solutions for this vulnerable population.

Following this, we developed 5 personas based on the categories and subcategories identified. This approach adheres to standard persona construction practices by using data-driven classification [28]. Moreover, the personas expand traditional persona content to include social dynamics, mHealth access, mHealth usage, mHealth interest, symptom diversity, symptom severity, specific needs, and design points to enhance richness in understanding older adult breast cancer patients and their context [32]. According to previous studies, the enriched insights gained provide valuable guidance for functional requirements, design, and implementation strategies tailored specifically for this target population [21]. The structure and content of our personas, which incorporate the key features of symptom management, can serve as templates for developing personas for other health consumer target populations [21].

We believe that the personas developed can effectively highlight both common and distinct traits among users. The shared characteristics of the 5 personas should serve as the primary focus for core functionalities, while the unique traits will inform the development of supplementary and optional features. From our research findings, we have initially identified 2 core functionalities: symptom reporting and educational materials.

The symptom self-reporting feature will enable users to log their symptoms daily, facilitating real-time monitoring and proactive management of potential side effects, which is crucial for breast cancer management for older adult patients [33]. For instance, for the Positive Manager, visualizing longitudinal symptom assessments could enhance their understanding of health trends over time. In the case of Dependent Parents, the mHealth product could facilitate a dyad approach where patients and their children both engage with the system, allowing symptom reports to be sent directly to their children via push notifications.

Meanwhile, the educational materials will provide tailored information to enhance users’ understanding of their condition and treatment, empowering them to make informed decisions regarding their health. For the Clinician-Dependent, it is crucial to integrate a voice icon and profile icon, enabling medical advice to be easily accessible and conveyed by a trusted physician. Regarding the educational features of the mHealth product, Positive Manager and Imanage Manager are inclined to learn and manage their health and could benefit from self-directed reading materials and a patient support network, while Clinician Dependent, who prefers individualized attention from health care providers, could be offered one-on-one video appointments. Given that the target population consists of older adult patients who often experience memory and comprehension difficulties, the development team must carefully manage the amount of information presented to avoid cognitive overload. A modular approach can effectively cater to users, providing essential knowledge for those who prefer not to delve deeper while allowing for broader exploration through optional modules. Active information seekers, such as the Positive Manager and Image Manager, can benefit from this modular design, which ensures continuous content updates and relevance.

As for the role of personas in the design process, they serve as educational tools for design and usability teams. They provide insights that convey project progress to sponsors and offer a “word picture” that familiarizes team members with target user groups. Personas structure user typification, enhancing communication within development teams. As noted by Hill and Barteck [34], once established, personas become the primary medium for design discussions, aiding in resolving group conflicts by referencing user perspectives. For instance, team members might ask, “What would the Clinician-Dependent think?” while designing the interface and algorithm. Additionally, based on interaction design principles like the double-diamond design, the iterative process often involves usability testing [35]. Personas can help predict who the primary and secondary users will be. For breast cancer patients, spouses and children are typically considered secondary users, as they can facilitate adoption and usage and are often willing to assist primary users in receiving better care. Therefore, detailed personas can serve as a foundation for selecting and screening participants for interactive prototyping and usability testing, directly influencing the solution’s design and development [21]. For instance, when considering Dependent Parents, it is crucial to involve their children as secondary users of the mHealth tool, as their experiences and insights are equally valuable.

During the implementation stage, personas serve as essential tools to guide the deployment of mHealth solutions. For the Positive Manager, promotional efforts such as posters and advertisements can effectively reach this group. For the Image Manager, word-of-mouth within social circles is a key avenue for adoption. The Clinician Dependent persona can be introduced to mHealth solutions through doctors or nurses during appointments, making it crucial to integrate mHealth into the clinical workflow. For the Dependent Parent, the focus can be on caregivers who accompany them to appointments, using this relationship as a key promotional touchpoint. Additionally, for long-term adherence, especially with Dependent Parents, their children should be considered the primary channel to improve compliance and ensure ongoing engagement with the mHealth tool.

According to previous studies, personas do not need to be recreated from scratch for every application [36]. Our 5 specified personas—The Positive Manager, The Dependent Parent, The Management Isolationist, The Image Manager, and The Clinician Dependent—can be reused by both researchers and practitioners. With China’s rapidly aging population and increasing prevalence of cancer, innovative approaches to patient management are urgently needed [37]. These personas offer valuable insights that can address the current gap in China’s user-centered design practice, particularly in mHealth solutions tailored to older adult breast cancer patients. Their reusability makes them adaptable for various health care applications, enhancing design efficiency and consistency across future projects.

### Limitations

Our study presents some limitations that should be acknowledged. First, we relied on interviews for data collection, without using focus groups. While one-to-one interviews provided in-depth insights into individual experiences, focus groups could have uncovered collective perspectives and group dynamics, particularly valuable when studying older adult populations. Second, the personas we developed are based on qualitative data, which, while rich in detail, are inherently subjective. The personas were constructed by researchers based on interpretations of the qualitative data, which may introduce some bias. Quantitative approaches to persona development, such as surveys or statistical analysis, could offer more objective, testable profiles that may provide additional validity and generalizability to the findings [38].

### Conclusions

This study identified symptom management behaviors and preferences for managing symptoms using mHealth among older adult Chinese breast cancer patients. Further, we developed 5 personas that reflect the diverse preferences of this population. These personas play a crucial role across multiple stages of mHealth development: helping to determine core functionalities during the planning phase, guiding design and development by aligning product features with user needs, and supporting implementation by facilitating adoption and engagement strategies. Together, these insights contribute to more effective, user-centered mHealth solutions.

## Abbreviations

mHealth: mobile health
